# A qualitative assessment of the health systems factors influencing the prevention of malaria in pregnancy using intermittent preventive treatment and insecticide-treated nets in Ghana

**DOI:** 10.1186/s12936-022-04159-w

**Published:** 2022-04-27

**Authors:** Virtue Fiawokome De-Gaulle, Joseph Kamgno, Verner N. Orish, Agnes Kotoh, Wilfred Mbacham, Harry Tagbor, Pascal Magnussen

**Affiliations:** 1 Faculty of Medicine and Biomedical Sciences, University of Yaoundé I, Yaoundé, Cameroon; 2 Faculty of Health and Medical Sciences, University of Copenhagen, Copenhagen, Denmark; 3 School of Medicine, University of Health and Allied Science, Ho, Ghana; 4 School of Public Health, University of Ghana, Legon, Ghana

**Keywords:** Malaria, Health system, Intermittent preventive treatment, Sulfadoxine-pyrimethamine, Insecticide-treated nets, Pregnancy, Ghana

## Abstract

**Background:**

Ghana has adopted and implemented intermittent preventive treatment using sulfadoxine-pyrimethamine (IPTp-SP) and insecticide-treated nets (ITNs) in an antenatal care (ANC) context to prevent malaria among pregnant women. However, the increased ANC attendance and its frequency facilitated by a free maternal health care policy in Ghana does not correspond with the uptake of IPTp-SP and ITN use among pregnant women. This study sought to elucidate the contextual health system factors influencing the delivery of IPTp-SP and ITN from a related quantitative study conducted in Ghana.

**Methods:**

This is the qualitative section of a mixed-methods study design, where audio recorded key informant interviews (KIIs) were conducted with health workers from across health facilities, districts, regional and national health directorates. The KIIs elicited information on health worker knowledge, perceptions, and rationale for the delivery practices of IPTp-SP and ITN revealed in the quantitative findings. The interviews were transcribed and imported into NVivo for analysis. Using the World Health Organization (WHO) Health Systems Framework as the theoretical basis, thematic analysis was conducted under broad themes of the building blocks. Findings are presented in narrative quotes, with a mindmap used to summarize the various health system factors and their interrelated relationship influencing the delivery of IPTp-SP and ITN.

**Results:**

Health system factors identified included health staff untrained on malaria delivery directives due to an ineffective trainer of trainer (ToT) system. Additionally, health worker confusion on when to commence SP (at quickening or ≥ 16 weeks) was found to result in delayed start of SP. Stock-outs in facilities due to procurement delays at the national level resulted in missed opportunities to deliver SP to eligible pregnant women at the ANC. Similarly, ITN stock outs led to eligible pregnant women not receiving one at ANC clinics.

**Conclusion:**

Poor health worker knowledge on policy directives, a consequence of ineffective training strategy led to delayed delivery of IPTp-SP to eligible pregnant women. Supply chain management challenges related to stock of SP and ITN resulted in missed opportunities to deliver the interventions to pregnant women attending ANC.

**Supplementary Information:**

The online version contains supplementary material available at 10.1186/s12936-022-04159-w.

## Background

Millions of pregnant women in sub-Saharan Africa (SSA) are exposed to malaria infection annually, especially *Plasmodium falciparum,* and this results in morbidity and mortality for both mother and baby [1–3]. In 2019, for example, one-third (11 million) of the 33 million pregnancies that occurred in the region were estimated to be exposed to malaria infection and this resulted in about 822,000 low birth weighted (LBW) babies [3]. Poor fetal growth due to interference of transplacental transport of nutrients to the fetus by parasitized erythrocytes sequestered in the placenta is what usually causes LBW attributable to malaria in pregnancy [4]. Maternal anaemia, a consequence of the destruction of red blood cells (RBC) by malaria parasites is one of the main effects to the mother [5, 6]. To prevent malaria in pregnancy (MiP) and its complications to both mother and child, the World Health Organization (WHO) recommended the use of intermittent preventive treatment with sulfadoxine-pyrimethamine (IPTp-SP) and distribution of insecticide-treated nets (ITNs) as part of antenatal care (ANC) [7]. These interventions have been made financially accessible to pregnant women through donor and governmental funding [8]. Hence, at no cost, all pregnant women must be given an ITN, and starting as early as possible in the second trimester, IPTp-SP must be administered under directly observed therapy (DOT), with at least three doses given at monthly intervals [7, 9]. More than 30 endemic countries in SSA, including Ghana, have adopted and implemented these interventions [3, 10]. However, coverage of at least IPTp-SP3, the least recommended dose and ITN is well below the target of 80% and 100%, respectively [3, 10]. As at 2019, IPTp-SP 1, 2 and 3 coverage in the African region was 62%, 49% and 34% respectively [3], and ITN ownership of at least one ITN per household was 68% [3], with only 52% of pregnant women reported to be sleeping under ITNs [3].

In Ghana, the MiP policy guideline specifies that every ANC registrant must be given an ITN and counselled on use [11]. In addition, IPTp-SP should be administered to pregnant women starting from 16 weeks of gestation or quickening, with monthly intervals for subsequent doses and administered under DOT [11]. A minimum of three doses is recommended during pregnancy, although the medication can be given till the time of delivery [11].

Despite increased ANC attendance and its frequency facilitated by a free maternal health care policy instituted in the country since 2008 [12], uptake of IPTp-SP and ITN use among pregnant women has been below the national target of 80 and 100%, respectively [13]. Between 2011 and 2016, fluctuating increases and decreases in IPTp-SP 1, 2 and 3 were recorded nationally. IPTp-SP 1, 2 and 3 uptake decreased from 69.7, 56.2 and 39.8% in 2011 to 53.4, 37.5 and 23.3% in 2014. These proportions increased slightly (69, 58 and 41.3%) in 2015 and decreased again in 2016 to a level below 2011 [14]. Although the 2019 malaria indicator survey (MIS) reports an increased uptake of IPTp-SP3 (61%), it is still below the national target [13]. The fluctuating and sub-optimal uptake of the intervention necessitates an assessment of the accountable factors. Uptake of this intervention could be influenced by a number of factors including individual factors such as pregnant woman’s acceptance or refusal based on personal choices such as perceived side effects [15] or delayed and infrequent ANC attendance due to financial constraints or lack of autonomy to seek health care without husbands consent [16–19].

In Ghana, Amankwah and Anto, revealed individual and health system related factors affecting uptake of IPTp-SP. Pregnant women who started ANC during their second trimester, and those who attended more than five times received more doses of IPTp-SP compared to those who started in their third trimester and less than five times attendances [20]. In addition, stock-out of SP attributed to the period when the national medical store got burnt and health worker nonadherence to the DOT strategy influenced uptake [20]. Health system factors directly linked to the delivery of the interventions have also been cited as a possible major cause [10, 16]. Factors such as limited health financing, inaccurate documentation of health records, and untrained health workers to effectively deliver interventions could affect the delivery of mip services, and result in the sub-optimal uptake. Weaknesses in the health system to effectively implement malaria interventions have been explained to be possibly attributable to the fact that investments in malaria control in endemic countries have mainly centred on procuring commodities, with little or nothing spent on addressing health system gaps impeding intervention coverage [10].

A mixed-methods study was conducted to assess the delivery appropriateness of IPTp-SP and ITN at ANC clinics [21] and also ascertain the health system factors influencing the delivery practices of the interventions. The quantitative section used a defined delivery algorithm based on the Ghana national MiP guideline to outline the steps or actions that need to occur for each intervention to be delivered appropriately or not [21]. While a higher proportion of pregnant women experienced appropriate delivery of IPTp-SP (76.3%) and ITN (58.6%), a significant percentage did not [21]. This qualitative study was conducted concurrently to elucidate the underlining contextual health system factors influencing the delivery practices observed in the quantitative findings. This study sought to understand how factors such as health worker knowledge, rationale, and higher health system factors including policy, supply chain management, and training influence delivery of IPTp-SP and ITNs to pregnant women attending ANC.clinics. Acknowledging the multisectoral nature of the health system as well as its interconnectedness for effective functioning, the WHO Health Systems Framework (WHO-HSF) [22] was used as the theoretical basis in order to evaluate the components of the health system. The six building blocks (service delivery, health workforce, information, medicines and products, health financing, and leadership and governance) in the framework, provided a measure by which the health system was assessed [22].

## Methods

### Study site

**Fig. 1:**
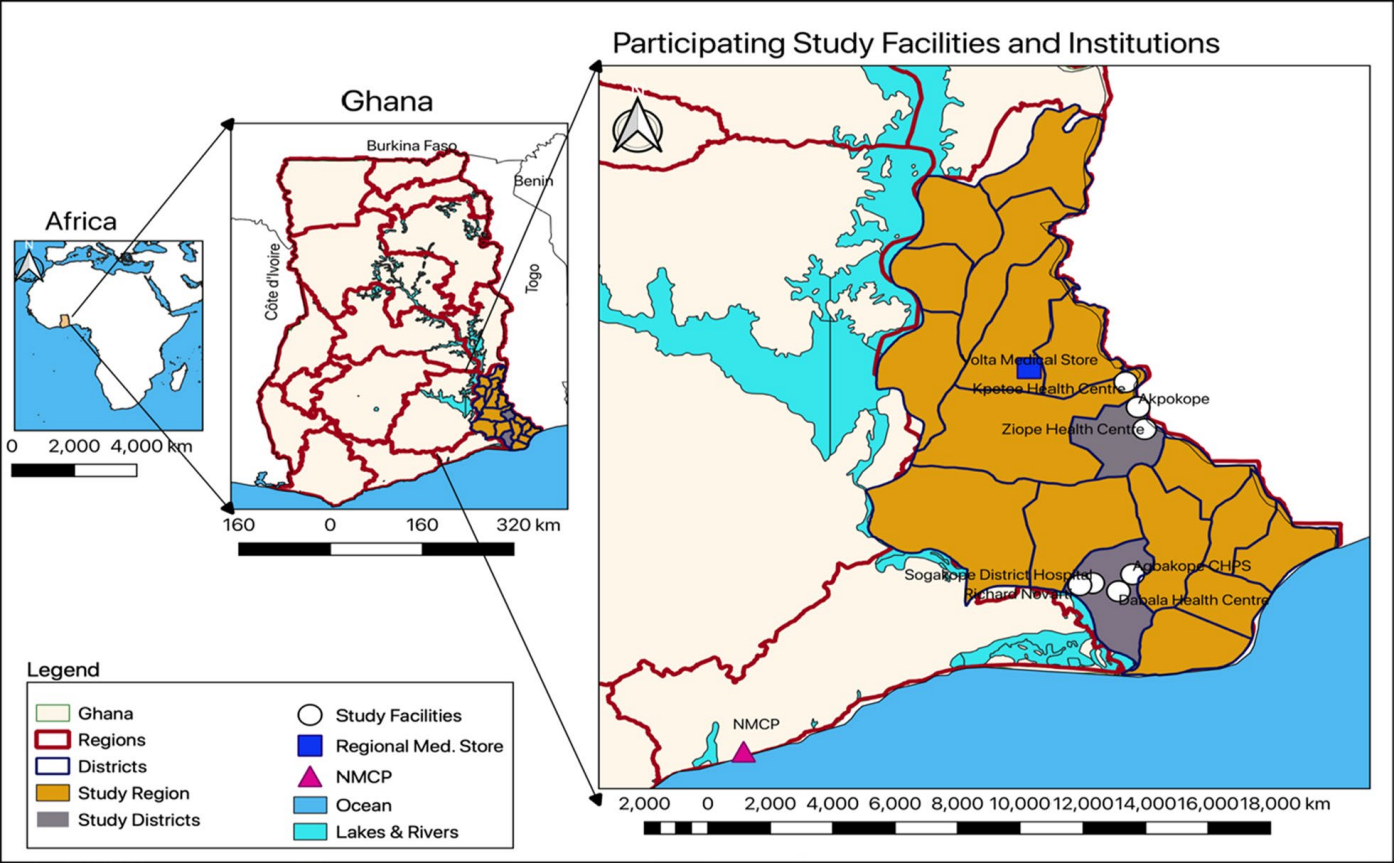
Study area

This study was conducted in Ghana, a West African country bordered to the west by Côte d’Ivoire, the north by Burkina Faso, the east by Togo, and to the south by the Gulf of Guinea [23] (Fig. 1). Based on inter census projections for 2020, Ghana has a population of about 31 million people, 51% of whom are females [24]. Malaria is holoendemic and perennial in all parts of the country, with the entire population at risk of infection [25]. About 90% of all malaria cases in Ghana are caused by *P. falciparum* parasites (most lethal) and transmitted by highly anthropophilic vectors, including species of the *Anopheles gambiae* complex and the *Anopheles funestus* complex [26]. The country is divided into 16 administrative regions with the Volta Region being one of them. Data was collected across four levels of the health system in the country, that is health facility, district and regional health directorates, and the national level (NMCP). Specifically, the study sites included seven health facilities, two district health directorates (South-Tongu and Agortime-Ziope), the Volta Regional Medical Store, and the NMCP.

### Study design

This study is the qualitative section of a mixed concurrent cross-sectional study carried out to assess the health systems factors influencing the delivery of IPTp-SP and ITN to pregnant attending ANC clinics. Data was collected over a period of five months: from April to June 2019 and from November to December 2019. This paper presents in-depth textual data on health system factors influencing the delivery of IPTp-SP and ITN. A phenomenological approach, which involves the study of the lived experiences of people in order to understand a particular phenomenon [27] was used. The experiences and practices of health workers in relation to the delivery of the interventions as well as other health system factors, including supply chain management of malaria commodities, health trainings, supervision, and other context-specific factors affecting delivery were elicited.

### Study population

The study population included health workers and managers at the facility, district, and regional health directorates as well as the NMCP to represent the views of the different hierarchical levels within the health system. In each facility, the ANC in-charges (mainly midwives) and their assistants were eligible to participate in the study. Furthermore, facility in-charges (physician assistants in health centres or medical directors for hospitals) were also eligible. At the district level, district health directors, district public health nurses, health information officers, and malaria focal persons were eligible. At the regional level, the regional malaria focal person and the medical store manager were eligible. Key personnel at the NMCP included focal persons for MiP, malaria data analyst, and deputy programme manager.

### Sampling and data collection procedure

Data for this study as well as the companion quantitative study were collected across three levels of healthcare delivery facilities; hospitals, health centres and Community-Based Health Planning Services (CHPS) Compounds. A detailed overview of the selection of the study facilities is presented in the published companion paper [21]. In summary, two hospitals, three health centres and two CHPS were sampled across the two health districts; South Tongu and Agortime-Ziope district.

Key informant interviews (KIIs) were conducted using open-ended interview guides structured under themes. The themes included: delivery practices of IPTp-SP and ITN, supply chain management of medicines, trainings on MiP, health worker knowledge on MiP policy directives, supervision and monitoring, health financing, and health data management. Although the guides were structured, relevant themes that emerged during the course of an interview were explored and factored into successive interviews that followed. The interview guides were designed and conducted in English. After conducting 30 KIIs, a level of saturation was reached (Table 1).

**Table 1:** Number of health workers and managers interviewed

Type of participants	Number of KIIs
*Health facilities*	
Midwives	9
Physician assistants	2
Physicians	1
Health information /records officer	4
Laboratory scientist	3
Sub-total	19
*District Health Directorate*	
District directors	2
District health information officers	2
District public health nurse	1
Sub-total	5
*Regional health directorate*	
Regional Malaria Focal Person	1
Regional Medical Store Manager	1
Sub-total	2
*National level (NMCP)*	
Deputy programme manager	1
National MiP focal person	1
Procurement and Supply Chain Management officer	1
Data management officer	1
Sub-total	4
Total KIIs	30

### Data analysis

All KIIs were audio-recorded using a digital voice recorder and transcribed verbatim into Microsoft Word by trained research assistants. The transcripts were further cross-checked with the audios by rereading it alongside listening to the audio recording by one of the authors (VFD). This was to ensure that the absolute content of the interviews were transcribed, and the original meaning of statements were maintained. To improve readability and increase comprehension, some edits were made to the transcripts, especially correcting misheard words and sentences with grammatical deficiencies.

Analysis of the qualitative data started with reading and re-reading of the transcripts to identify themes relating to the various aspects of the health system factors influencing the delivery of IPTp-SP and ITN. A theme refers to a specific pattern found in the data [28]. The development of the themes for this analysis was done deductively and inductively. Deductive methods of theme generation entail the use of existing theoretical concepts to formulate themes [28], hence, using the health system building blocks to formulate the main themes was a deductive method of theme generation. The main themes developed were on; leadership and governance, medicines and products, service delivery, health information and workforce factors influencing the delivery of IPTp-SP and ITN. Using inductive methods of theme generation, where the themes are identified from within the data [28], sub-themes were developed through reading and rereading of the transcripts. A codebook was then developed to outline the various themes, their operational definition, and examples of statements that should be considered for coding under a particular theme. The codebook was used as a guideline to create nodes within the NVivo Version 12 software for qualitative analysis.

The transcribed interviews were imported into the software. Each transcript was read line by line, with relevant statements coded under related nodes, respectively. The various nodes were analysed into themes to reflect the content of the data. Thematic analysis identifying common patterns or ideas [28] was carried out. Thus, recurrent patterns on how IPTp-SP and ITN are delivered as well as the recurrent contextual factors such as accessibility of medicines, supervision, health training, and policy factors affecting delivery of the interventions were identified. The results of the qualitative data analysis are presented in narratives and supported with illustrative quotes from study participants. To triangulate and explain the quantitative findings in the companion paper with the qualitative data, significant quantitative findings are presented in the results and discussion sections with the reference article cited. A summary of all the identified health system factors, and their interrelatedness in function is presented graphically with a mindmap (Wondershare Edraw MindMaster).

## Results

### Context within which IPTp-SP and ITN is delivered

Thirty KIIs were conducted, including four from the NMCP, two, from the Regional Health Directorate, five from across the two districts and 19 from the seven health facilities (Table 1). The years of experience in the respective roles of the participants ranged from four to twenty-two years. All the study facilities had National Health Insurance Scheme (NHIS) accreditation, hence, pregnant women with valid NHIS cards do not pay for maternal health care services including ANC. First time ANC attendees without a valid NHIS card, were asked to go and register for the insurance after a confirmatory pregnancy test.

The district health directorate, managed by the District Health Management Team (DHMT) provide supervisory and management support to the health facilities within their health area as well as public health services. Specific to malaria control, the district malaria focal person and or the district public health nurse engage in supervision, monitoring and coordination of malaria control activities including trainings. The regional malaria focal person coordinates all the malaria control programmes in the region and liaises with the districts directorates to supervise the delivery of malaria control services, ensure distribution of commodities like mosquito nets and rapid diagnostic tests (RDTs) and coordinate malaria trainings. Medicines and medical commodities are stored at the Regional Medical Store (RMS) and distributed directly to facilities under the management of the regional medical store manager. A distribution mechanism, termed the ‘Last Mile Distribution’ (LMD) is used in Ghana, where medical commodities are distributed from the regional medical store to every level of healthcare facility. The NMCP coordinates all malaria control programmes nationally including policy formulation and its dissemination, training of relevant health personnel, sourcing of funds to procure malaria commodities, supervision, and monitoring to ensure adherence to policy directives.

### Health system factors influencing the delivery of IPTp-SP

**Fig. 2:**
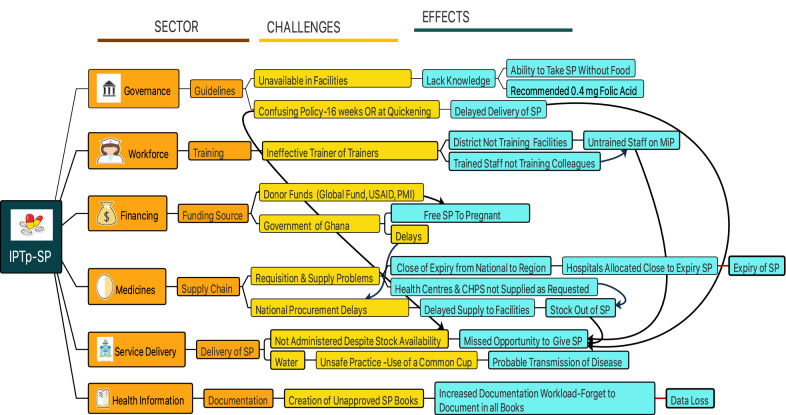
Summary of health system factors influencing the delivery of IPTp-SP

The actions and inactions at every level of the health system; national, regional and district had direct effect on the delivery of the interventions to pregnant women at the ANC clinic. An interconnected relationship between the various facets of the health system, including governance, health workforce, health financing, medicines and products, and data documentation were found to influence the delivery of IPTp-SP and ITN in health facilities (Fig. 2) (Additional file 1).

### Governance

The availability and accessibility of policy guidelines to health workers is one of the ways to ensure health worker awareness and knowledge of recommended delivery practices to enhance appropriate delivery of IPTp-SP. This study revealed the unavailability of MiP policy guidelines in three out of seven study facilities as illustrated in the quotes by ANC health staff.

“No, I have not seen any policy guideline and don’t know if there is one in the ANC or the facility” (Female midwife, 4 years in ANC, IDI, Health Centre -1).

“We don’t have the guideline for malaria in pregnancy, what we have is the guideline for case management, but they said it should be at the in-charges office (health facility in-charge), when you need it, you go there” (Female midwife, ANC in-charge, 11 years in ANC, IDI, Health Centre -2).

While it is important to formulate policies and make it accessible to health workers, it is further imperative to train these healthcare workers on these policies, thoroughly explaining the recommended delivery practices including the rationale for such directives. Frontline health workers in this study were split into two groups. Whilst some revealed participating in malaria trainings, others had never attended any malaria training or in-service training since they started working in the ANC units.

“Yes, I have gone for a malaria training recently, about three months ago. It was a national programme. It was on malaria in pregnancy, IPTp, case management and all that. It was quite intensive, but it is worth it” (Female midwife, 4 years in ANC, IDI, Hospital -2).

“No, I have not gone for any malaria training since I started working here (4 years), and nobody has done any internal or in-service training to train me or any of the nurses here on malaria” (Female midwife, 4 years in ANC, IDI, Health Centre -1).

“In my 22 years of work as a health worker, I have never been to any malaria or HIV training and I am always coming into contact with blood” (Female midwife, 22 years in ANC, IDI, Health Centre—1).

To understand the reason for the dual findings on training, interviews at the NMCP elicited the training strategy employed, and it was revealed that a trainer of trainer (ToT) system is used to train health workers on different thematic areas on malaria including MiP. In attendance are health professionals and managers from regional and district health directorates, who are tasked to in turn organize trainings for health workers in the various facilities within their health area.

“When it comes trainings at the NMCP, we have national facilitators who are experts in the various fields. MiP is usually done alongside other areas, such as case management, data and diagnostics, procurement and supply chain management. We ensure the training information reflects what we have in the policy guidelines after which we decide on the various categories of staff to be trained, like midwives, health information officers, malaria focal person, biomedical scientist and pharmacists. A ToT is then organized for regional and sometimes district facilitators to go and ultimately train the services providers” (National MiP Focal Person, KII, NMCP).

Dissemination of the knowledge and skills acquired through the ToT to all health workers in the facilities within a particular district is hinged on trained district personnel organizing training sessions for facility level health workers. The trained health workers are also expected to pass on the knowledge and skills to their colleagues who could not attend these organized training sessions. However, communication gap between district health directorates and health facilities were revealed to impede this training. An interview with the regional malaria focal person revealed how health workers in some districts are not aware of the policy directives related to IPTp-SP delivery, although personnel at the district level have been trained and expected to disseminate it to all relevant staff.

“We at the regional level cannot reach all facilities, that is why we have district officers. You know at the DHMTs level you don’t provide service, you are only to ensure that the people on the ground are doing the right thing. So, for instance if in your district you have 10 midwives, what stops you from visiting these 10 midwives or even calling them for a half day meeting to go over a two sheet directive that has come. but they don’t do some of these things” (Regional Malaria Focal Person, KII, GHS).

### Health workforce and service delivery

Poor knowledge on policy directives related to the delivery of IPTp-SP was therefore one of the emergent themes. Incorrect interpretation of policy directive on when to start IPTp-SP emerged as a predominant theme. Most of the health workers across the various levels of health facilities interpreted the Ghana policy directive of starting ‘IPTp-SP at 16 weeks of gestation OR quickening’ [11] as starting IPTp-SP at ‘16 weeks of gestation AND quickening’. Emphasis was mostly laid on the experience of quickening, rather than known gestational age of 16 weeks. Pregnant women, mainly between 16 to 20 weeks of gestation who have not experienced quickening or could not tell if they have, were more likely to miss their first IPTp-SP dose. Similarly, findings from the quantitative study revealed that pregnant women between the gestational ages of 16 to 20 weeks, recorded the least odds of appropriate delivery despite stock availability [21]. Health workers explained how they differ the commencement of the medication to their next scheduled ANC visit (usually a month away) on the basis that quickening has not occurred, irrespective of a known gestational age of 16 weeks and above. The following quotes illustrate these points:

“The SP, that one, they give at quickening. Well, it is quickening, and some also say 16 weeks, but we normally give it at quickening because it is not everybody that feels the baby movement at 16 weeks” (Male physician assistant, 6 years as PA, IDI, Health Centre -3).

“If they have not experienced any movement or quickening, we leave them. You ask them do you hear the movement once a day, twice a day or just a slight movement and it stops, some will say me I just hear it quick and it will stop, so for me when I hear them say that I don’t give it to them because I am not sure if quickening has begun, so I give it the next month, even if she is 16 weeks, me I still don’t give them because the client can spot” (Female midwife, 4 years in ANC, IDI, Health Centre -1.)

“Most at times, especially the primips, there are instances where they are 16 weeks but say they haven’t experienced quickening, in that case I personally, I prefer them coming back. So, the next visit, then from there we give. You know the primips they feel quickening by 18 weeks there about, so by the time they come back for their next visit they are ready to take it. (Female midwife, 4 years in ANC, IDI, Hospital -2).

Health worker knowledge on the recommended dose of folic acid to be taken concurrently with SP, without compromising its effectiveness revealed another knowledge gap. The 2014 Ghana MiP policy state 0.4 mg folic acid as the recommended dose that can be taken concurrently with SP [11]. An update in the policy indicated the use of iron preparations containing not less than 60 mg elemental iron and not more than 1.5 mg folic acid, a composition that fits iron (iii) polymaltose as the routine haematinic to be given when SP is started [29]. This was not commonly known to most of the health workers, as they incorrectly explained that 5 mg folic acid can be taken concurrently with SP, but taken a few days later. The following quotes support these assertions:

“Normally, if you are taking the SP, you don’t add the folic acid to it immediately, I mean the 5 mg folic acid, because it doesn’t go with the SP. So if they take the SP, we tell them to start the 5 mg folic acid the following day. I learnt the SP has some effect on the folic acid, so if you take the folic acid with the SP immediately, it wont work.” (Female midwife, ANC in-charge, 12 years in ANC, IDI, Health Centre-2).

“The folic acid is given whether first trimester, second trimester or third trimester on a daily basis. But I learnt when you give the SP the client is not supposed to take the folic acid that very day but the following day. A senior staff also said it should be five days later, but me I don’t know, because I haven’t gone for any training on it. I learnt the client will have some rashes on the skin and the eyes will become red” (Female midwife, 4 years in ANC, IDI, Health Centre -1).

### Medicines, products and financing

One of the key determinants of the delivery of SP as an IPTp is its availability in health facilities. Issues with supply chain management of SP spanning from the national level to the facility level were found to affect the delivery of the intervention. These included stock-outs, and drug requisition and distribution challenges between health facilities and the regional medical store. SP is procured at the national level, with funding from a number of stakeholders including the Government of Ghana (GoG), and donor agencies such as the Global Fund (GF) and the United States Agency for International Development- Presidents Malaria Initiative (USAID-PMI) There are times when the procurement of SP is undertaken by the either the Global Fund, USAID-PMI or the GoG as illustrated in the quotes below.

“Government of Ghana procures SP as its contribution or counterpart funding to the Global Fund. In fact, Global Fund is supposed to be complimentary but invariably, the chunk of the money has been coming from the Global Fund and the other donors. Then government to show her commitment procures SP or some other funds are allocated for health worker salaries and/or health infrastructure” (Key Personnel-1, KII, NMCP).

While securing funds is essential to facilitate purchasing of the medicine, ensuring timely procurement, is imperative to ensure its availability in the health facilities. The procurement process for medical commodities including SP involves a number of steps bound by specific timelines. A delay at any point in this process invariably affects the stock levels of the medicine in the country, and likely to result in stock-out. KIIs at the NMCP revealed procurement delays as the cause of stock-out of SP in the country in 2019 (period of data collection).

“Last year (2019), the government was supposed to procure, it didn’t come at the time that we had agreed it should come, I don’t know what had contributed to that delay, but you know the procurement system, you call for tenders, you open, then evaluate and you award contracts, the supplier then delivers. All these are guided by specific mandatory time frames. But it does happen that once in a while at one point or the other you get delays. And when the SPs are delivered, the food and drugs authority, test for the quality, when the quality doesn’t measure up, they quarantine it and reorder the supplier to deliver a different batch altogether. These are some of the reasons why the SP batch for last year (2019) was delayed, because they did not deliver it on time and the quality analysis also took some time” (Key Personnel-1, KII, NMCP).

“Delays in arrival of the medicine in-country is what leads to stock-outs. Lets say GoG is supposed to procure, and by your estimation the goods are supposed to be in-country by maybe March of this year, and the goods delay, you are expecting a stock-out. So, we do know ahead of time that we are approaching stock-out and sometimes you cannot do anything about it, because you are waiting and the thing is not in-country, how do you distribute it?” (Key Personnel-2, KII, NMCP).

The trickle effect of this stock-out at the national level invariably affected stock availability at regional medical store and subsequently, the health facilities.

“We’ve had issues of stock-out consistently. We are getting issues of stock-out as we are trying to improve access for the stock to get to as many facilities as possible. We continue to have issues of stock-out between national and region, and region and facilities. So, these are issues that we experience and we address almost every day. We get calls of stock-out of SPs in facilities, clients come and go without getting the SP so they miss their IPTp dose” (Regional Malaria Focal Person, GHS, KII).

“Currently we don’t have SP and the reason is that there is a delay in procurement. The SP hasn’t arrived at the national level so we have exhausted what we have here” (Regional malaria focal person, GHS, KII).

Health facilities, mainly the health centres and CHPS compounds narrated their experiences of stock-out of SP, a situation leading to missed opportunities to administer the medication to eligible pregnant women. The quantitative finding also revealed how approximately 37% of the eligible pregnant women who were not offered IPTp-SP occurred during periods of stock-out.

“During the second quarter of this year, between April to May (2019), I run out of stock of SP, it was not in the district at all, I think for about one to two months. If the SP is not there, I don’t prescribe for them (pregnant) to go and buy, so that time we were not having I didn’t dispense to them, so they missed their medications” (Female midwife, 10 years in ANC, IDI, CHPS Compound-1).

“Hmmmm even SP kroaa (kroaa, a local expression meaning “even the basic”) we don’t have. Our facility is not having SP, we borrowed from other facilities, because when they (RMS) brought the medicines none of the SP was inside. So, we went to (facility name removed) to ask for some. and that one too is finished, so the pregnant women don’t get” (Female midwife, ANC in-charge, 10 years in ANC, IDI, Health Centre—3).

Contrary to the experiences of stock-out at the health centres and CHPS compounds, the hospitals reported constant supply of SP.

“Never, we have never run out of stock of SP, we always have more than enough every time” *(Male Pharmacist, 17 years as pharmacist, IDI, Hospital—1).*

*“I can say we have not run out, I have never experienced stock-out of SP, no” (Male Pharmacist, 5 years as pharmacist, IDI, Hospital—2)*

Rather, they experience overstocking and this sometimes leads to expiration of the medicine in their stores. This situation was explained to be due to close to expiry drugs being supplied to them by the RMS, when they already have enough and have not placed a requisition.

“We have expired SP. You know, we did not request for it and they (RMS) brought us an allocated one, so that was the main reason. Because it was an allocation, we cannot reject it, we have to take it. Mostly, what I have realized is that, when they are doing allocation, then it means the dates for the product is not good at the regional level. So normally when those ones come, we push the ones with the closer expiry date, and if we are not able to consume it before time, it expires” (Male Pharmacist, 5 years as pharmacist, IDI, Hospital—2).

“So, like I said you will be there and they will allocate to you, if we were the ones requesting, we would have requested based on our consumption specifically. Because, sometimes consumption patterns also change, so normally we calculate for every six month to know the current consumption of the item. So, I presume that that the SP they sent us, the expiry date was not long enough” (Male Pharmacist, 17 years as pharmacist, IDI, Hospital – 1).

Probing on circumstances that lead to close to expiry drugs being allocated to hospitals from the RMS, the medical store manager explained that he also receives close to expiry drugs from the national level. The Ghana Health Service (GHS) and Ministry of Health (MoH) were blamed for this as they are the ones in charge of procuring and authorizing distribution. Hence, delayed distribution of medicines or procurement of close to expiry drugs is their fault as explained in the quote below.

“Sometimes we get the quantity of SP alright but we are afraid of the expiry date that we will receive it and it will expire on us because we get close to expiry products. If any blame it will have to go to the national level Ghana Health Service, Ministry of Health because they procure. The IHS (imperial Health Sciences) are just logistics management people, they are not the ones who purchase the medications. They store and transport it, so if the Ghana Health Service say move, they move it they only advice the day it will expire or finish” (Regional Medical Store Manager, GHS, KII).

### Service delivery

The strategy of administering SP under DOT requires the availability or provision of safe drinking water. Care must be taken not to expose pregnant women to other infections through unsafe practices such as using a common cup or unclean water. While almost all the study facilities were observed to comply with the DOT strategy, with women either purchasing a sachet of water or bringing their own water to take SP, one health centre placed a cup on a bucket of water for pregnant women to use to take the SP. This practice was acknowledged as unsafe and unhealthy by an ANC staff in the centre, however, for fear of being tagged as the junior staff instituting a business of selling sachet water to pregnant women, she has decided to keep mute on the practice.

“I came and met it like that, the ANC in-charge here, they fetch water and they use the same cup to drink the water if they have to take the SP. My question is who buys the water for them to take? Do you buy it and put it here and sell it to them at 20 pesewas? and tell them (pregnant woman) buy water, take your SP? Or what? if someone doesn’t have money, are you going to dash the person? Or is the money for the water going to come from the facilities coffers, who is buying the water? they use the same cup to fetch water and take the SP since I came but that is what they do here. Me, the junior, I will not say that let us buy pure water (sachet) and put it here and sell to the pregnant women before they say that it is (Ama-pseudo name) who is introducing business in the room, no!” (Female midwife, 4 years in ANC, IDI, Health Centre -1).

### Documentation

Evidence of delivery of any intervention requires proper documentation. To ensure this, parameters to be documented as well as documenting tools are fundamental. In Ghana, although IPTp-SP can be taken up to the time of delivery [11], for quantification purposes, the limit for delivery in the health facilities has been capped at 5 doses across all health facilities as explained by personnel at the NMCP in the quote below.

“For now we are advocating for 5 doses to be given to the pregnant women. We are also advocating that for the pregnant women in Ghana they start taking SP, starting at 16 weeks and then they take it till delivery just that sometimes for quantification purposes we need to peg it at a number, so that is why we peg it at 5 doses” (Key Personnel-3, KII, NMCP).

The ANC register is the standard tool used for documenting all services offered to pregnant women and this includes documentation of IPTp-SP doses given. Details about the women when she first attends ANC are captioned in a particular section. During her subsequent visits, the health worker turns to the page on which the woman was registered to fill out the routine services delivered to her including doses of IPTp-SP administered. At the end of each month, the number of SP doses given, and other services are retrieved from the ANC register to fill monthly report forms. In the CHPS compounds and some health centres with limited number of ANC clients, health workers complied with this procedure as illustrated below:

“We document the SP here, this is the ANC register, so we have a part for documenting SP, so this is what we use” (Female midwife, 3 years in ANC, IDI, CHPS Compound—2).

“We use the ANC register to document the SP” (Female midwife, 10 years in ANC, IDI, CHPS Compound-1).

“We fill the SP section in the ANC register” (Female midwife, ANC in-charge, 10 years in ANC, IDI, Health Centre—3).

Sometimes because of the workload, if want to tally in another book and you forget, it will not work well, but by all means you will write it here, once you are writing the thing against the persons name you will fill everything. If you tally, l think is secondary work” (Female midwife, ANC in-charge, 10 years in ANC, IDI, Health Centre—3).

However, in hospitals and some of the health centres with large number of ANC clients, flipping the pages of the ANC register to document SP given at each visit was described as cumbersome. Additionally, retrieving data from the ANC register on the number of SP doses administered in a month to fill monthly report forms was described as difficult. Health workers in these facilities explained how they have improvised by creating “SP books” using either exercise or notebooks. This, they explained eases the process of flipping the pages of the ANC register to retrieve data on the SPs administered, especially to fill their monthly report forms.

“We have a separate book where we document the SP, we designed it for easy access when it comes to the report collation, even though there is a section in the ANC register, we want to make it easy when it comes to the report collation” (Female midwife, 4 years in ANC, IDI, Hospital -2).

“We have the SP book where we fill the IPTp, we write the clients name, the dose, that is first dose, or second and we tally. So for the IPTp book, we total the number of pregnant women who have taken the first dose, the second dose, we total it for the month and we don’t have to go to the ANC register to count the number of pregnant women who took the first dose, second or third to fill our reports” (Female midwife, 4 years in ANC, IDI, Health Centre -1).

Some health workers explained that the creation of “SP book” different from the ANC register increases their workload, because it increases the number of books and registers to fill. An instance was given of how IPTp-SP given to a pregnant woman was not documented in the SP book. This was explained to result in data losses, since they use the records in the improvised SP books to fill the monthly report forms.

“I will say it is making the work so difficult, the writing to me personally is too much, because in the ANC register we have the IPTp column, so when it happens like that you will see that by all means something is left out, I may enter the IPTp-SP given in the ANC register and forget to write it in SP notebook we have created, so sometimes clients come with their ANC card and I have documented that she has been given the IPTp-SP and the client confirms being given, but it is not documented in the SP book. I would say we are making the work difficult, you write here and you write here, the same thing” (Female midwife, 4 years in ANC, IDI, Health Centre -1).

This improvised practice of SP books, although known at the national and regional level, is not endorsed, because of its likelihood to result in data loses. Especially, when they are unable to update the standard source document, which is the ANC register.

“We are against separate improvised tally books and the use of exercise book. Some midwives like to buy this school children’s exercise book and when they give SP they will be writing it in it. It was resulting in data losses” (Regional Malaria Focal Person, GHS, KII).

To help abort this unapproved practice, the national level (NMCP) designed a tally sheet to help ease the documentation process and monthly reporting of IPTp-SP. Through the regional malaria focal persons, the designed tally sheets are to be distributed to the health facilities with health workers trained on how to use it.

“The NMCP around 2016/2017 initially developed a soft copy of a tally sheet and the regional focal persons were supposed to train, best to my estimation, all health workers should at least be aware of it” (National Malaria Focal Person, MiP, NMCP, KII).

“We are only encouraging the use of ANC registers and the SP tally sheet that the programme (NMCP) has introduced, the national level designed an SP tally sheet which we have distributed” (Regional Malaria Focal Person, GHS, KII).

Despite the design and onward distribution of the tally sheet, coupled with training of district officers to disseminate as explained by the regional malaria focal person, the practice of documenting SP in the improvised SP books still continues. The communication gap between the district level health system and the health facilities was cited again as the reason some of the facilities are not aware of the SP tally sheet recommended by the NMCP.

“We can’t say all the facilities have it (SP tally sheet)), it also comes back to district officers not ensuring it gets to the facilities, because practically, we at the regional level cannot reach all facilities, that is why we give it to district officers so that when they go they can also give it to their facilities within the district that are providing ANC services (Regional Malaria Focal Person, GHS, KII).

### Health system factors influencing the delivery of ITN

#### Medical products

Stock-out of ITN was the main factor identified as influencing the delivery of ITNs. ITNs, just like SPs are procured at the national level and distributed across the country. However, due to its bulkiness, the nets are not stored at the regional medical stores, but distributed to the districts, where they are stored in the district medical stores for onward distribution to the health facilities.

“We take ITNs from national but because of its bulkiness, it does not come to the regional medical stores. The nets go straight to the district from the national. This started about 3 or 4 years ago so we don’t receive nets at all, we don’t have space for them” (Regional Medical Store Manager, KII, GHS).

Health facilities make their requisitions for ITNs needed through the district health directorates. Requisition are made to last a period of three months, however, health workers explained that requested quantities are not met, with less supplied.

“For the ITN, we collect it from the DHD direct, when they started, I have been collecting 150, that is for 3 months. But it reached a point, if you write the 150, they will give you 50, that means every month I have to request for it. So, every month I have to be going to the district to go and collect them” (Female midwife, ANC in-charge, 12 years in ANC, IDI, Health Centre-1).

“The ITN is requested from the district directorate. When you request too, the last time we requested 100, they gave us 50, so it’s like that” (Male Physician Assistant, A, 1.5 years as PA, IDI, Health Centre—1).

Inability to supply requested quantities to health facilities was revealed to be due to limited supply at the district level as quoted below:

“Hmmm usually they do the distribution at region, so the distribution can be done based on the quantity of the nets that they have received, so at times you can do the allocation and the possibility that they can’t get all is there. We are into the supply chain so it happens. You will bring your request, maybe 20, but based on the stock at hand you will be given 5 or 10” (Store keeper, 10 years experience, IDI, Hospital—1).

Health workers in almost all the facilities reported stock-out of ITN within the last 12 months, and stock-out also observed during the data collection period. This results in some eligible pregnant women missing the opportunity to get an ITN during an ANC visit as revealed in the quantitative findings, where about 21% of eligible pregnant women who were not given an ITN occurred during periods of stock-out [21]. In the health centres and the CHPS compounds, health workers explained that stock-outs were either due to unavailability of the net in the district or delay in the district transporting requested ITNs.

“I have told the district our nets are finished and the storekeeper said the pickup truck will bring it but they have not brought it. See, the pickup has just come here but did not bring the nets. Me, I will sit here and wait for the nets, I will not go and carry nets here in a trotro (mini bus used for public transport)” (Female midwife, ANC in-charge, 12 years in ANC, IDI, Health Centre-1).

“I think the beginning of the year we run out. But even it didn’t keep long before they (district) brought us some. So, like six to five people didn’t get it, the district wasn’t having some” (Female midwife, 10 years in ANC, IDI, CHPS Compound-1).

Contrary to health workers recounting experiences of stock-outs and its observation during the data collection period, interview with one of the regional level managers revealed rarity of ITN stock-out. He explained that even if it happens, it could be due to lack of proactiveness between the districts and the facilities, or challenges in transporting the nets from the districts to the facilities.

“No, ITN is one commodity that we don’t run out of, that does not happen, but if it does at the facilities, then it is proactiveness between the district and health facility. There are some facilities that don’t have motorbikes and there have been instances where facilities were not given transport like TnT, so the ITNS were lying at the DHMT (district) stores and facilities were not having it. So we have sent out a directive to all DHMTS that they should ensure that they use the DHMT pickup truck to always pick the nets and distribute to the various facilities that need them, but if there is any CHP zone that has the motor bike and wants to pick, they can pick it” (Regional Malaria Focal Person, GHS, KII).

## Discussion

This study sought to assess the contextual health system factors influencing the delivery of IPTp-SP and ITN to pregnant women as revealed in the companion quantitative study [21]. Furthermore, qualitative means of enquiry enable the collection of in-depth data on people’s understanding, perception and experiences. Hence, this study sought to also unearth health system factors across the various hierarchical levels to get a holistic understanding of factors influencing delivery of IPTp-SP and ITN to pregnant women in Ghana. Using constructs from the WHO Health System’s Framework [22], the findings and discussions are structured under the building blocks.

The qualitative findings revealed an interconnected relationship between various sectors of the health system and how they affect the delivery of IPTp-SP and ITN. The first of which is health worker confusion about the policy directive on when to initiate IPT-SP. Whilst the Ghana policy directive states that IPTp-SP should be started at ‘16 weeks of gestation **OR** quickening’ [11], health workers across the various levels of health facilities interpreted it as ‘16 weeks of gestation **AND** quickening’. Emphasis was laid on the experience of quickening, rather than on gestational age. This resulted in the first dose of the prophylactic treatment differed to the next scheduled ANC visit, usually a month apart. This practice was observed at all the levels of the health care facilities and even among health workers who had attended malaria training including IPTp-SP. This finding explains the quantitative data, where pregnant women between the gestational ages of 16 to 20 weeks had the least odds of appropriate delivery [21]. This is because pregnant women unable to perceive quickening despite being in the eligible gestational age might not be given the medication due to the health worker misinterpretation of the policy directive. Most ANC health workers also had incorrect knowledge on the appropriate dose of folic acid to be taken concurrently with SP, while district level managers knew the correct dose. This underscores the lack of, or ineffective training and poor monitoring and supervision of health workers at the ANC. The ineffective malaria trainings could be because MiP is usually a sub-section of trainings as revealed in a KII at the NMCP, and therefore too little time is allocated to ensure that pertinent delivery directives are comprehensively explained. Another reason for the incorrect or poor knowledge of health workers on MiP delivery directives could be the inherent limitation of the ToT strategy. The ToT strategy is hinged on transferring information from one person to another, where there could be distortion of information [30], especially, if training manuals are not made available to the district level managers to use as reference materials for health workers. Health workforce factors related to lack of knowledge of policy directives resulting in missed opportunities to deliver IPTp-SP to pregnant women have been reported in a number of studies [23–26]. In Mali, health workers withheld SP from pregnant women in their ninth month of pregnancy, because they said it could cause birth deformities including heart problems, despite their policy guideline specifying the medication should be given up to delivery [34]. Training of health workers on policy guidelines must not only be limited to informing them of what the guidelines says, but also explain the rationale behind. The African region has the largest shortages of health workers [35], with the ratio of nurses and midwives to a 10,000 people being 9.8 in 2018 [36]. In Ghana, that ratio is 42 per 10,000 people [36]. It is, therefore, imperative to ensure optimal performance of the limited health workforce through effective trainings which will equip them with the requisite knowledge and skills to meet up the health needs of the people.

Another health system factor found to predominantly affect the delivery of IPTp-SP was the stock-out of SP in the health facilities, mainly the health centres and CHPS compounds. While the quantitative paper reported this occurrence and provided the proportion of inappropriate delivery that occurred during those periods, it did not present the underlining cause of the stock-out. Findings from this qualitative paper revealed the cause to be due to logistical gaps at the national level, where governmental commitment to procure the medication was not prioritized. Reasons for the delay were in two-fold, one, the procurement of the medicine delayed, and two, the inability of the procured medicines to meet the quality check at the Ghana Food and Drugs Authority (FDA). Health financing, especially for the procurement of medicines and medical products is one of the significant pillars in the provision of care. Thus, governmental commitments should not only be promised, but consistent and timely to avoid stock-outs. Stock-outs of medical commodities due to delays in funding and quality control checks can be avoided if proper quantification is done at the national level. Because, the NMCP should be cognisant of these instances over the years and factor them in the computation of the national minimum and maximum stock levels of SP, so that these delays do not affect stock levels in the country. Pregnant women in this study missed the opportunity to receive SP during scheduled ANC visits due to stock-outs, with almost 37% of eligible pregnant women who were not offered the medication occurring during periods of stock-out in the quantitative study [21]. Unlike in other countries, where health workers prescribe SP for pregnant women to buy outside health facilities in times of stock-out [37], in Ghana, SP use is restricted to IPTp and IPTi, which are to be administered in health facilities. Health workers, therefore, do not prescribe it for the women to purchase outside the ANC in pharmaceutical or drug stores. This means that access to the medication is impeded until the facility stock is replenished.

While some studies have reported non-compliance with the DOT delivery strategy, with the most cited reason being unavailability of water [30–33], the contrary was observed in this study with health workers enforcing the DOT strategy. Pregnant women either bought sachet water sold for 20 Ghana Pesewas (0.035$) or came along with their own water to take the medication.

Insecticide-treated nets, the second preventive intervention was observed to be out of stock in one hospital and two health centres out of the seven study facilities during the data collection period, although only for a short time. In the hospital, the stock-out lasted for two days, and in the health centres, it lasted for about 6 days. Stock-outs at the health centre level was attributed to delay in the district transporting requested quantities to them in a timely manner, whereas in the hospital, it seemed to have been due to delayed requisition by the health workers because they were observed to get a restock the next day from the district. This goes to underscore the response from the regional malaria focal person who explained that ITNs are always in stock, with stock-outs in facilities most likely due to health workers not requesting for it. Stock-out of ITNs at ANC clinics leading to pregnant women not receiving one or deferring receipt till stocks are available is a common occurrence, as reported in the companion quantitative study and other studies conducted in African countries [21, 31, 34, 35]. While ITN ownership does not necessarily translate into use, its availability coupled with continued counselling on the significance of use for malaria prevention could influence behaviour and attitude in-terms of use. Hence, adequately training medical store managers and storekeepers as well as effective monitoring and supervision could minimize the stock-out situation due to health workforce factors.

This study has strengths and some limitations. The use of qualitative methods for assessing health worker practices, their perceptions and reasons for what they do, enabled the in-depth collection of data, highlighting health system gaps to explain the quantitative findings and also identify other factors including ineffective ToT system of training, supply chain management gaps and health financing challenges affecting the delivery of IPTp-SP and ITN in Ghana. Qualitative research inherently has some limitations including bias of the researcher in the interpretation of findings, usually described as a subjective interpretation of findings. While acknowledging this inherent limitation, this study aimed at interpreting the findings using established constructs from the health systems building blocks as a guide. This, therefore, limited the researcher and interviewer bias in the interpretation of the findings.

## Conclusion

In conclusion, an interrelated network of health system factors was found to influence the delivery of IPTp-SP and ITN to pregnant women attending ANC clinics. Poor health worker knowledge on policy directives, a consequence of unavailability of policy guidelines in facilities, ineffective ToT strategy and poor supervision of service delivery at the ANC was revealed. Supply chain management challenges stemming from delayed procurement of medicines at the national level led to stock-outs of SP at the facility level and subsequently missed opportunities to deliver the interventions to pregnant women attending ANC. The DOT delivery strategy was practiced in almost all the study facilities. ITN stock-outs at the facilities was due to delayed transportation of requested stocks from the district health directorates to CHPS compounds and health centres.

## Supplementary Information

**Additional file 1.** Interview Guides.

## Data Availability

The dataset analysed for this study is available from the corresponding author on reasonable request.

## Abbreviations

ANC: Antenatal care
CHPS: Community-Based Health Planning Services
DHMT: District Health Management Team
DOT: Directly observed therapy
GHS: Ghana Health Service
LBW: Low birth weight
LMD: Last mile distribution
IPTi: Intermittent preventive treatment for infants
IPTp-SP: Intermittent preventive treatment for pregnant women using sulfadoxine pyrimethamine
IDIs: In-depth interviews
ITNs: Insecticide-treated nets
MiP: Malaria in pregnancy
MIS: Malaria Indicator Survey
MoH: Ministry of Health
NHIS: National Health Insurance Scheme
PSM: Procurement and Supply Chain Management
SP: Sulfadoxine-pyrimethamine
SSA: Sub-Saharan Africa
RMS: Regional medical store
ToT: Trainer of trainer
WHO: World Health Organization
WHO-HSF: World Health Organization Health Systems Framework
