# Supplementary material for: A qualitative assessment of the health systems factors influencing the prevention of malaria in pregnancy using intermittent preventive treatment and insecticide-treated nets in Ghana
Source: Malar J. 2022 Apr 27;21:136. doi: 10.1186/s12936-022-04159-w (PMC9044599; doi:10.1186/s12936-022-04159-w)
Supplement: Supplementary file 1 — Additional file 1. Interview Guides. [file 12936_2022_4159_MOESM1_ESM.docx]

**Key Informant Interview Guide**

**Assessing health system effectiveness in the control of malaria among pregnant women in the Volta Region of Ghana**

**Interview with National Malaria Control Programme (NMCP)**

**Region_____________________________________________________________________**

**Title and qualification of the person interviewed__________________________________**

**Years of working at NMCP___________________________Years in post___________**

1. Please tell us what is the role of the NMCP?
2. What are the main interventions for controlling malaria among pregnant women in this country?
3. What is the funding mechanism for acquiring malaria commodities? Probe on government subvention? Global Funds?

**IPTp-SP**

1. Kindly explain the rationale for administering IPTp-SP to pregnant women in Ghana?
2. Please explain the policy directive on administering it? Probe on when it should be commenced, ask about 16 weeks or 13 weeks and the concept of quickening.
3. Kindly tell me about the DOT strategy of administering SP. Probe on its practice in facilities.
4. Probe on the national standard on the number of doses to be given maximum during pregnancy (3 doses, 4 doses or 5). Probe on reason for that cut-off.
5. How would you describe the uptake of SP against ANC attendance, Probe percentage of uptake. Probe on ANC attendance and SP uptake disparity
6. With regards to IPTp-SP, kindly explain the funding and supply to facilities. Probe on if IPTp-SP is given to be given free of charge or at a cost.
7. Are there instances, where funding for the procurement of the drugs is delayed and that leads to stockouts? Probe what is done in such situations and how it affect the implementation of the intervention.
8. Please can you explain to us the supply chain of IPTp-SP to facilities? Probe on distribution to regional medical sotre, mode of distribution, cost of transportation borne by facility or who?
9. How is the quantification of IPTp-SP and ITN done in order to meet the national demand? Probe on the reason why there is a current stock out of SP and ITN in facilities.
10. How would you describe the stock availability of SP in health facilities in the country? Probe on what the NMCP is doing about the stockout situation of the medicine.
11. What would you say is the cause of stockout of the SP to be given free of charge to the pregnant women in the health facilities in the country?

**ITNs**

1. Kindly explain the policy on ITN in Ghana with regards to pregnant women attending ANC clinics.
2. What is the ITN stock availability stock situation in Ghana for the ANC clinics? Probe on stock availability in the past two years in facilities.
3. So now, what is the way forward with ITN stock in the facilities for the pregnant women and children under two years?

**Training of health workers**

1. Kindly explain how training health workers on malaria in pregnancy is done in Ghana.Probe on frequency funds for trainings and source of funding (government or donor funds). Probe on the concept of trainer of trainers (ToT)

**Supervision**

1. Kindly describe the situation of supervision and monitoring of delivery of MiP services in the ANC units in the country. Probe of frequency, funds availability and source of funding for supervision.

**IS THERE ANYTHING YOU WOULD LIKE TO ADD TO THIS CONVERSATION?**

**Thank you for your time!**

**Key Informant Interview Guide**

**Assessing health system effectiveness in the control of malaria among pregnant women in the Volta Region of Ghana**

**Interview with Regional & District Malaria Focal Person/ District Health Director**

**Region_____________________________________________________________________**

**Title and qualification of the person interviewed__________________________________**

**Years of working at institution ________________________Years in post___________**

1. Kindly tell me what you do as the regional/district malaria focal person/district director of health?
2. How would you describe the prevalence of malaria among pregnant women in this district/region, (probe on increase or decrease of malaria cases over the past one year)?
3. Kindly tell me about the IPTp-SP situation in this district. Probe on main suppliers, either regional medical or NGOs (stock levels).
4. When IPTp-SP is available, what mechanisim is used to distribute it to the various facilities? Probe on how quantities supplied to the facilities in this district. Are pre-defined quantities given or they are based on their consumption pattern?
5. Kindly tell me about uptake of preventive malaria interventions in the district (Probe ANC attendance (over 80%) in relation to uptake of low IPTp-SP (39%) and ITN uptake).
6. Tell me about the ITN situation in this district. Probe on availability.
7. What is the procedure for obtaining or receiving ITN for this district from (Regional or district medical store). Probe command issuance or administrative procedure.
8. How does the district deliver these supplied ITNs to the facilities? probe on if district transports it to the facilities itself bearing the transport cost or if it is transported to facilities at a fee? Probe on if facility picks up ITNS themselves.
9. Does the district have enough storage capacity for supply of ITNs? Probe on how the storage capacity affects the necessary quantity needed to supply to all the health facilities in this district.
10. Does the district supply IPTp-SP, ITN and RDTs to all the facilities under it irrespective of whether they are public, private or confessional?

**MiP Trainings and supervision**

1. Are MiP trainings organized for health workers in this district? Probe on when the last session was organized.
2. How often are the work activities of the health facilities in this region supervised and monitored (Probe on specific supervision done, where and when?)

**Policy on malaria**

1. What is the current policy on administering IPTp (probe on use of gestational age or quickening, ability to take it on an empty stomach, the dosing of folic acid recommended to take with IPTp-SP)
2. How accessible are the policy document to the health workers? (ask for a copy of the policy)

**Data management**

1. Is malaria one of the tests pregnant woman do when they come for their first ANC? Or must pregnant women be screened for malaria on their first ANC visit? Probe reason if yes or no
2. Where must pregnant women who come for to the hospital with clinical malaria be treated? Probe if at the ANC OR OPD?
3. If at the ANC, where is it documented? Is there a standard book for documenting it ? If at the OPD, where is it documented?
4. Where are the health information officers supposed to take the MiP data for their monthly reporting?
5. How often do you organize trainings for the health information officers on malaria health records?

**IS THERE ANYTHING YOU WOULD LIKE TO ADD TO THIS CONVERSATION?**

**Thank you for your time!**

**Key Informant Interview Guide**

**Assessing health system effectiveness in the control of malaria among pregnant women in the Volta Region of Ghana**

**Interview with National/ District / Facility Health Information Officer**

**Region_____________________________________________________________________**

**Title and qualification of the person interviewed__________________________________**

**Years of working at institution___________________________Years in post___________**

**Information about health worker**

1. Before we start the interview, kindly tell me a few things about yourself. Please what is your specialization?
2. How many years have you been working as a district health information officer?

**Health records documentation in Ghana**

1. Kindly tell me what you do as theNational/ district/facility health information officer?
2. Describe how health services delivered at the health facilities are reported to the district. Probe on monthly return forms.
3. How is IPTp-SP uptake/ coverage reported from the facility to the district?
4. How are health records recorded and reported at the national level? Probe into if they use the district health information system (DHIS). If DHIS is used, who is responsible for entering the data into the system? If DHIS is not used, how is health data documented and reported?

**Documentation of clinical malaria in pregnancy cases**

1. How is the data on clinical malaria cases reported to the district?
2. How do you get that data?
3. Is malaria one of the tests pregnant woman do when they come for their first ANC? Or must pregnant women be screened for malaria on their first ANC visit? Probe reason if yes or no
4. Where must pregnant women who come for to the hospital with clinical malaria be treated? Probe if at the ANC OR OPD?
5. If at the ANC, where is it documented? Is there a standard book for documenting it ? If at the OPD, where is it documented?
6. Where are the health information officers supposed to take the MiP data for their monthly reporting?
7. How often do you organize trainings for the health information officers on malaria health records?

**Data management**

1. How is data managed in lower level health facility, (probe on the health information officers at the health centres and the CHPS compounds).

**IS THERE ANYTHING YOU WOULD LIKE TO ADD TO THIS CONVERSATION?**

**Thank you for your time!**

**Key Informant Interview Guide**

**Assessing health system effectiveness in the control of malaria among pregnant women in the Volta Region of Ghana**

**Interview with ANC Health Staff / Lab Scientist**

**Region_____________________________________________________________________**

**Title and qualification of the person interviewed__________________________________**

**Years of working at institution ________________________Years in post___________**

**Information about health worker**

1. Before we start the interview, kindly tell me a few things about yourself. Please what is your specialization? Are you a midwife?
2. How many years have you been working as a midwife/General nurse? How many years have you worked in the ANC?
3. How many years have you worked here in the facility’s ANC unit?

**Case management among pregnant women**

1. Now lets talk about pregnant women, when do they usually start coming for ANC? Probe, if they come early, what are the reasons OR if they come late, what are the reasons
2. Generally, do the pregnant women come for their scheduled visits you give them?
3. So when a pregnant woman comes to the ANC for the first time, what is done for her? Probe the labs requested for and ask about malaria tests.
4. If malaria test is done for first-timers, ask the reason why it is done?
5. Do you sometimes get pregnant women with positive malaria results even though they did not come with any complaints or symptoms? In such instances what is done for them? Are they given treatment?
6. Ok, so when pregnant women come with symptoms suggestive of malaria, how are they managed? Probe on tests conducted, and treatments given in the first and second trimester.

**Insecticide treated Nets**

1. What does the Ghana policy say on giving mosquito nets at the ANC unit?
2. Who is supposed to supply the facility with the nets? Probe on regional medical store of district stores?
3. Do you currently have mosquito nets in this ANC given to the pregnant women free of charge?
4. If they are out of stock, for how long has this been so? Have you requested for it and you have not been given?
5. In your estimation, would you say the pregnant women who come here have mosquito nets they are sleeping under or they do not have?

**Intermittent preventive treatment with Sulfadoxine Pyrimethamine**

1. How does this facility get supply of SP, does it come free from the government or the facility buys it?
2. Do you have stock of SP from the government or the district?
3. If SP is out of stock in the facility, what is done? Is it prescribed for them to buy? Probe on cost is yes.
4. Tell me, if the pregnant women has to go and buy it and take, do you think they buy it and take it?
5. Tell me, what is the Ghana policy on giving IPTp-SP? Probe on when pregnant women can start taking it (Gestational age requirement or quickening)
6. If the pregnant woman is 16 weeks but she has not experienced quickening, can she be given the IPTp-SP?
7. How must IPTp-SP be taken by pregnant women, can it be taken on an empty stomach? Are the pregnant women supposed to take the IPTp-SP infront of the health worker? Or can they take it home?
8. Is the IPTp-SP taken under DOT in this facility when there is stock at the ANC? If it is not taken under, why is that so?
9. How many doses of fansida must a pregnant woman take during pregnancy?

**Cost of ANC services**

1. What is the cost of ANC services for the pregnant women? Probe on First time attendee costs and continuing clients.
2. What is the cost of managing a case of clinical malaria among pregnant women.

**Training of ANC staff on IPTp-SP or malaria case management**

1. Have you gone for any training on malaria among pregnant women in the last two years? Have you been trained on IPTp-SP and the management of malaria in pregnancy.

**Supervision**

1. Have you had any supervisory visit from the district, regional or national level on how you deliver care to pregnant women with regards to malaria?

**IS THERE ANYTHING YOU WOULD LIKE TO ADD TO THIS CONVERSATION?**

**Thank you for your time!**
